# Natriuria and calciuria levels in preeclampsia: a cross-sectional study

**DOI:** 10.1590/S1516-31802013000100021

**Published:** 2013-04-01

**Authors:** Rose Gasnier, Edimárlei Gonsales Valério, Janete Vettorazzi, Sérgio Hoffmeister Martins-Costa, Elvino Guardão Barros, José Geraldo Lopes Ramos

**Affiliations:** I MD. Gynecologist and Obstetrician, Hospital de Clínicas de Porto Alegre (HCPA), Department of Obstetrics and Gynecology, Universidade Federal do Rio Grande do Sul (UFRGS), Porto Alegre, Rio Grande do Sul, Brazil.; II MD. Gynecologist and Obstetrician and Professor, Hospital de Clínicas de Porto Alegre (HCPA), Department of Obstetrics and Gynecology, Universidade Federal do Rio Grande do Sul (UFRGS), Porto Alegre, Rio Grande do Sul, Brazil.; III MD, PhD. Nephrologist and Professor, Hospital de Clínicas de Porto Alegre (HCPA), Department of Obstetrics and Gynecology, Universidade Federal do Rio Grande do Sul (UFRGS), Porto Alegre, Rio Grande do Sul, Brazil.

**Keywords:** Pregnancy, high-risk, Pre-eclampsia, Hypertension, pregnancy-induced, Natriuresis, Diagnosis, differential, Gravidez de alto risco, Pré-eclâmpsia, Hipertensão induzida pela gravidez, Natriurese, Diagnóstico diferencial

## Abstract

**CONTEXT AND OBJECTIVE:**

Sodium excretion abnormalities in preeclampsia have been studied in relation to several factors. The objective of this study was to compare natriuria (mEq/24 h) and calciuria levels (mg/24 h) in preeclamptic patients.

**DESIGN AND SETTING:**

An analytical cross-sectional study with a control group was conducted in the obstetric center and the high-risk pregnancy outpatient clinic at a university hospital in southern Brazil, and in a primary healthcare unit in the same city, including pregnant women with mild preeclampsia, severe preeclampsia or chronic hypertension, and women with normal pregnancies (14 patients in each group).

**METHOD:**

Natriuria was measured using an ion-selective electrode in an automated clinical chemistry analyzer (Hitache 917, Roche). All the patients collected 24-hour urine, at home or at the hospital, for analysis of proteins, creatinine, calcium, sodium and uric acid. Quantitative variables with asymmetrical distribution were described using the median, minimum and maximum, and were compared using the Kruskal-Wallis test. The results were logarithmically transformed, with one-way analysis of variance (ANOVA) by ranks and then the post-hoc Tukey test, and were analyzed by means of the Spearman correlation and receiver operating characteristic (ROC) curve. The significance level used was 0.05.

**RESULTS:**

There were significant differences between the groups in comparing severe preeclampsia with chronic hypertension and severe preeclampsia with controls (P < 0.0001 for both measurements). **CONCLUSION:** Natriuria levels may be lower in preeclampsia when associated with calciuria. Natriuria assessment is an additional test for differential diagnosis of hypertensive diseases in pregnancy, but is a poor predictor when used alone.

## RESUMO

**CONTEXTO E OBJETIVO:**

Alterações na excreção de sódio têm sido estudadas na pré-eclâmpsia relacionadas a vários fatores. O objetivo deste estudo foi comparar natriúria (mEq/24 h) com os níveis de calciúria (mg/24 h) em pacientes pré-eclâmpticas.

**ESTUDO E LOCAL:**

Estudo transversal analítico com grupo controle foi realizado no Centro Obstétrico e no Ambulatório de Gestação de Alto Risco em um hospital universitário no sul do Brasil, e na Unidade Básica de Saúde na mesma cidade, incluindo gestantes com pré-eclâmpsia leve e grave, hipertensão crônica e gestações normais, com 14 pacientes em cada grupo.

**MÉTODO:**

A natriuria foi dosada através da medida de íon eletrodo seletivo, utilizando analizadores automáticos de química clínica (Hitache 917 Roche). Todas as pacientes coletaram urina de 24 h, em casa ou no hospital, para análise de proteínas, creatinina, cálcio, ácido úrico e sódio. As variáveis quantitativas com distribuição assimétrica foram descritas por mediana, mínimo e máximo, e comparadas por teste Kruskal-Wallis. Os resultados foram transformados logaritmicamente, com ANOVA *one-way* por ranking e, posteriormente, teste *post-hoc* de Tukey, e foram analisados por médias de correlações de Spearman e curva ROC (*receiver operating characteristic*). O nível de significância adotado foi de 0.05.

**RESULTADOS:**

Foram encontradas diferenças significativas entre os grupos quando comparados pré-eclâmpsia grave com hipertensão crônica e pré-eclâmpsia grave com controles (P < 0.0001 para ambas as medidas).

**CONCLUSÃO:**

Natriúria pode estar reduzida na pré-eclâmpsia quando associada com calciúria. Avaliação da natriúria é um teste adicional para diagnóstico diferencial de doenças hipertensivas na gestação, mas é um pobre preditor quando utilizado isolado.

## INTRODUCTION

Preeclampsia is one of the major causes of maternal morbidity, preterm birth, intrauterine growth restriction and perinatal mortality.^1^^,^^2^ Its pathophysiology has been extensively studied, and its etiology is probably multifactorial. The disease is characterized by volume contraction, intravascular coagulation and vasoconstriction. It was previously thought to be triggered by an overactive renin-angiotensin-aldosterone (RAA) system, but studies have shown that the system is more complex than this. There is an inverse relationship between the plasma-active renin to prorenin ratio and the clinical severity of preeclampsia.^3^

Natriuretic factors also appear to be altered in preeclampsia. Several studies have reported increased atrial natriuretic peptide (ANP) in preeclampsia, but this is not a uniform finding.^4^ This event can precede the clinical emergence of the disease. In addition, changes in cell sodium transport are likely to accompany hypertension-induced pregnancy.^5^ In a case-control cross-sectional study, Reis et al. demonstrated that aggravation of hypertension in preeclampsia correlates with serum atrial natriuretic peptide (ANP) and brain naturiuretic peptide (BNP) concentrations, although BNP values may be influenced by the existence of a prior hypertensive state.^6^

Some studies have found reduced natriuria in preeclampsia, probably related to the hypocalciuria process. Because of renal involvement, reabsorption of sodium linked to calcium in the ascending loop of Henle has been described.^7^ McGrowder demonstrated a significant difference in natriuria levels between preeclampsia and normal pregnancies: 100.43 ± 16.61 for preeclampsia, 106.46 ± 14.98 for chronic hypertension, and 144.42 ± 16.37 mEq/24-hour for normotensive patients.^8^

## OBJECTIVE

The aim of our study was to evaluate the relationship between hyponatriuria and preeclampsia, and the possibility of its use, in combination with calciuria measurement, for preeclampsia diagnosis and to differentiate the forms of hypertension in pregnancy, by evaluating the natriuria and calciuria levels in pregnant women with chronic hypertension or preeclampsia and in normal controls.

## METHODS

An analytical cross-sectional study with a control group was performed, in which the factors evaluated were calciuria and natriuria in relation to preeclampsia. The patients selected were women in their 20^th^ to 37^th^ week of pregnancy, between March 2008 and November 2009. They were divided into four groups: severe preeclampsia, mild preeclampsia, chronic hypertension and normal pregnancy. The preeclamptic subjects were recruited at the obstetric emergency clinic of a university hospital located in the south of Brazil, upon hospitalization. The group of hypertensive women was recruited at the high-risk pregnancy outpatient clinic at the same hospital. The control group was normotensive, with no history of preeclampsia or hypertension in previous pregnancies, and was recruited at a primary healthcare unit.

The group with chronic hypertension and the normotensive controls collected a 24-hour urine pool at home, unlike the patients in the preeclampsia group, who were hospitalized. The patients were instructed to collect urine for 24 hours, starting with the second morning urine, until the first urine of the next day, thus completing 24 hours. The patients were asked to store the urine in plastic bottles in the refrigerator and deliver it to the laboratory at the end of the collection period. The collections from hospitalized patients were made in the same way and then sent for analysis.

The exclusion criteria for cases and controls were malnutrition, previous or gestational diabetes, renal diseases, previous significant proteinuria, superimposed preeclampsia, continuous use of calcium supplements or calcium channel blockers, drugs that alter sodium levels, major fetal malformations, intrauterine fetal death and multiple pregnancies.

The criteria used to diagnose diabetes followed the recommendations of the Fourth International Workshop Conference on Gestational Diabetes Mellitus.^9^

The criteria adopted for diagnosing preeclampsia (systolic arterial blood pressure ≥ 140 mmHg and/or diastolic arterial blood pressure ≥ 90 mmHg in two measurements, separated by 6 hours, with ≥ 300 mg/24 hours proteinuria). For classifying preeclampsia as severe, the criteria were those presented by the United States National High Blood Pressure Education Program Working Group (NHBPEP) in 2000.^10^

The patients’ 24-hour urine pools were needed in order to analyze their protein (mg/24 hours), creatinine (mg/24 hours), calcium (mg/24 hours), uric acid (mg/24 hours) and sodium levels (mEq/24 hours). In order to guarantee adequate 24-hour urine pooling, creatinine was assayed in the pool (24-hour urine pools with less than 600 mg of creatinine were discarded).^11^ After preeclampsia had been diagnosed, laboratory tests were carried out to assess its severity.

This study was approved by the Ethics Committee (project 07-563), and written informed consent was obtained from each subject before she joined the study protocol.

The laboratory tests were done at the clinical pathology laboratories at the same hospital. Natriuria was measured using an ion-selective electrode in an automated clinical chemistry analyzer (Hitache 917, Roche), and was expressed as mEq/l.

To calculate the sample, since we did not find any published report on this sodium analysis alone, the study by Ramos et al.^12^ on calciuria was used. In that study, the preeclampsia group showed calciuria of 82 ± 15.1 mg/24 h and the control group showed calciuria of 317 ± 86 mg/24 h,^12^ with 0.01 alpha and 0.90 beta risks. The number of patients calculated as necessary in each group was 14.

The quantitative variables with asymmetrical distribution were described in terms of the median, minimum and maximum, and were compared by means of the Kruskal-Wallis test. Next, the results were logarithmically transformed, with one-way analysis of variance (ANOVA) by ranks and then a post-hoc Tukey test. The results were analyzed by means of the Spearman correlation and the receiver operating characteristic (ROC) curve. The significance level used was 0.05. The database was built in Excel, and the analyses were done using the Statistical Package for the Social Sciences (SPSS) 16.0 software.

## RESULTS

The characteristics of the patient population at the time of inclusion in the study are presented in Table 1. Maternal age was statistically greater in the group with chronic hypertension than in the other groups. Fetal gestational age was significantly greater in patients with mild preeclampsia than in those with chronic hypertension. Mean arterial pressure was significantly higher in the group with severe preeclampsia than in the other groups. There were more primigravidae in the preeclampsia groups than in the other groups.

**Table 1.** Characteristics of the study groups

Characteristics	Severe PE (n = 14)	Mild PE (n = 14)	Chronic hypertension (n = 14)	Control group (n = 14)	P
Maternal age (years)	26.42 ? 6.03^a^	26.23 ? 5.03^b^	33.06 ? 6.44^abc^	26.71 ? 6.34^c^	0.008
BMI (kg/m^2^)	31.36 ? 6.80	31.07 ? 3.57	32.07 ? 3.55	29.49 ? 4.59	0.536 (NS)
GA (weeks)	30.25 ? 3.43	32.54 ? 3.02^a^	26.21 ? 6.40^a^	29.05 ? 5.39	0.010
MAP (mmHg)	122.47 ? 15.18^abc^	106.26 ? 10.51^ab^	102.40 ? 8.77^ac^	80.93 ? 11.86^abc^	0.0001
Nullipara	9 (64.3%)	7 (53.8%)	2 (14.3%)	3 (21.42%)	0.01
PE history	3 (21.4%)	2 (15.4%)	4 (28.6%)	0 (0%)	0.709 (NS)

Quantitative variables for symmetrical distribution are expressed as mean ? standard deviation, compared using one-way ANOVA and, subsequently, Tukey’s test. Categorical variables are expressed as n (%) and were compared using the chi-square test.

NS = not significant; PE = preeclampsia; BMI = body mass index; GA = gestational age; MAP = mean arterial pressure. ^abc^ = is an indication of which group is different in relation to the others.

The calciuria and natriuria levels can be seen in Table 2, showing progressive hypocalciuria and hyponatriuria from normal pregnancy to severe preeclampsia. The differences in these levels between patients with severe preeclampsia and chronic hypertension and between patients with severe preeclampsia and normal pregnancy were statistically significant (P < 0.0001).

**Table 2.** Natriuria and calciuria levels

	Severe PE (n 14)	Mild PE (n 14)	Chronic hypertension (n 14)	Control group (n 14)	P
24-hour urinary sodium (mEq)	109^ab^	166	203^a^	206.5^b^	0.043
	(198-932)	(15-343)	(91-346)	(88-568)	
24-hour urinary calcium (mg)	81.5^ab^	118	226^a^	272^b^	< 0.0001
	(3-164)	(8-564)	(50-162)	(60-489)	

Quantitative variables for asymmetrical distribution are expressed as median and minimum-maximum, calculated using the Kruskal-Wallis test, with subsequent logarithmic transformation ranking for one-way ANOVA and then the Tukey post-hoc test. PE = preeclampsia; ^abc^ = indication of which group is different in relation to the others.

**Figure 1.**
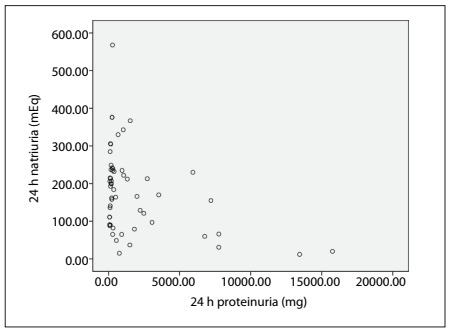
Proteinuria and natriuria correlation.

**Figure 2.**
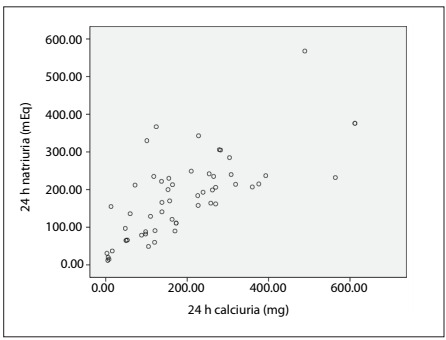
Calciuria and natriuria correlation.

The correlation between natriuria/proteinuria and natriuria/calciuria can be seen in Figures 1 and 2, respectively. The Spearman correlation was 0.734 between calciuria and natriuria.

**Figure 3.**
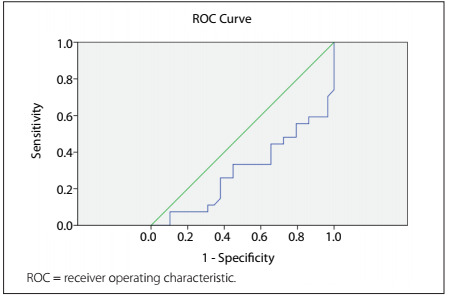
Natriuria and preeclampsia diagnosis.

The ROC curve demonstrated concordance between the sensitivity and specificity of 24-hour natriuria and the preeclampsia diagnosis, with an area under the curve of 0.841 (P = 0.0001), as seen in Figure 3. The best cutoff point was 177 mEq, showing sensitivity and specificity of 67%.

## DISCUSSION

Prediction of preeclampsia has been based on detection of risk factors and measurement of arterial pressure, proteinuria and edema. However, some pregnant women without risk factors will develop preeclampsia, thus demonstrating the necessity for biochemical markers that could predict this condition. The importance of predicting which women will develop preeclampsia lies in the need for special medical care and preventive measures that might prolong the pregnancy and reduce the maternal and fetal risks.^13^ The purpose of our study was to assess the correlation between natriuria and preeclampsia, and to evaluate the possibility of using this in the differential diagnosis of hypertension in pregnancy, in combination with calciuria levels. The classical methods of differential diagnosis between chronic arterial hypertension and preeclampsia work well, but biochemical analysis on urine could make a contribution in difficult cases, towards diagnoses that are more complex and accurate, thereby adding to knowledge of the renal pathophysiology of pregnancy-induced hypertension.

Our observation that preeclamptic patients present hypocalciuria is in agreement with reports from other investigators.^14^^,^^15^^,^^16^^,^^17^^,^^18^ These authors suggested that urinary calcium excretion levels may serve as a diagnostic tool for differentiating between the various forms of hypertension in pregnancy. This might be explained by an increase in tubular calcium reabsorption, rather than a decrease in glomerular filtration, since similar creatinine clearance values were found among the groups.

Frenkel et al. compared natriuria among preeclampsia, chronic hypertensive and normotensive patients and found values of 142 ± 42, 130 ± 35, and 122 ± 38 mEq/l, respectively, with no significant differences among the groups.^17^ Halhali et al. studied preeclampsia and normotensive groups, and showed natriuria levels of 87 ± 31 and 90 ± 29 mEq/l, which were non-significant values.^18^ Likewise, according to Selly et al., urine sodium excretion did not differ between groups (preeclampsia group with 124 ± 13 and normotensive group with 100 ± 8 mmol/24 hours).^19^

There are few studies on renal management in preeclampsia other than in relation to proteinuria. Ultrastructural glomerular alterations were demonstrated in one study: subendothelial deposits and fusion of podocytes were the most common features, and fusion of podocytes was correlated with the level of proteinuria.^20^ One important point has been the differential diagnosis with other forms of hypertension. Natriuria measurement, just like hypocalciuria and uric acid measurement, can help in this differentiation. Recent studies have demonstrated that the glomerular lesions present in preeclampsia could be related to placental antiangiogenic protein factors, but tubular function is not altered, as shown in the present study.

The strong point of this study is that it may lead the way towards a new kind of research on preeclampsia, especially with regard to analysis on medications for treating gestational hypertension. On the other hand, the small number of patients and the need for studies in other places with the same effect could represent weak points of this investigation. Other authors did not find any significant difference, but they also did not control for renal function or collect data on proteinuria and creatinuria levels.

One of the early events in preeclampsia may be excessive expansion of the extracellular fluid volume, which causes circulation of factors that modify the remodeling of the decidual vasculature, thus preventing normal placentation.^21^ Many studies have suggested that the plasma volume is lower in preeclampsia than in normal pregnancies. However, serum sodium concentration does not differ.^22^ A reduced glomerular filtration rate would contribute towards the reduced rate of sodium excretion in preeclamptic patients. Recent studies have shown that autoantibodies against the angiotensin II type 1 (AT1) receptor are present in the serum of preeclamptic patients. In cases of preeclampsia, compared with normal pregnancies, it was found that the AT1 receptor gene was upregulated fivefold in the decidua.^23^ It has been suggested that a maternal autoantibody with the ability to activate AT1 receptors might be implicated in the renal damage in preeclampsia.^24^

The kidneys have an important role in regulating blood pressure, extracellular fluid volume, sodium balance and water excretion. Glomerular filtration rate and renal plasma flow increase by 40% to 65% and 50 to 85%, respectively, during normal pregnancy. Increased renal perfusion pressure (RPP) causes a potent natriuretic effect (pressure natriuresis) that is greater in situations of hypertension and during pregnancy.^25^ Our results showed hyponatriuria in preeclampsia, rather than the natriuretic effects of progesterone, arginine vasopressin, atrial natriuretic factor, prostaglandins and other factors that may lead to excessive loss of filtered sodium.

Digitalis-like cardiotonic steroids may be involved in the pathophysiology of preeclampsia, as shown by observations that Digibind (a digoxin antibody) lowers blood pressure in patients with the disease.^26^ Adair et al. showed that there was significantly lower erythrocyte sodium-pump activity in severe preeclampsia than in normotensive pregnancy, and suggested that the plasma levels of biologically active endogenous digitalis-like factors (EDLF) are elevated in patients with severe preeclampsia.^25^

Current research is more strongly in favor of cell sodium alterations, perhaps mediated by circulating sodium-pump inhibitors, often leading to increased cell sodium. Increased cell sodium in vascular tissue has been shown to enhance vascular sensitivity to vasoconstrictor agents or lead directly to increased vasoconstriction, which causes elevated pressure levels. The low sodium content in extracellular tissues may have caused the hyponatriuria seen in our study.

The pathophysiology of natriuria and the levels to be considered require further studies before this criterion can be used in clinical practice. Such studies may help towards developing new medications for preeclampsia management, since there are insufficient reports in the literature to define this. The home collection of urine by some patients may have affected our results, even though the collections were controlled for creatinine levels, as described above, because of evidence that sodium, uric acid and water renal management may be abnormal in the final stages of pregnancy, depending on patient rest.

## CONCLUSIONS

In conclusion, natriuria levels may be lower in preeclampsia when associated with calciuria. This forms an additional test for the differential diagnosis of hypertensive diseases in pregnancy, but it is a poor predictor when used alone.
